# Jealousy in Dogs

**DOI:** 10.1371/journal.pone.0094597

**Published:** 2014-07-23

**Authors:** Christine R. Harris, Caroline Prouvost

**Affiliations:** University of California San Diego, La Jolla, California, United States of America; University of Tasmania, Australia

## Abstract

It is commonly assumed that jealousy is unique to humans, partially because of the complex cognitions often involved in this emotion. However, from a functional perspective, one might expect that an emotion that evolved to protect social bonds from interlopers might exist in other social species, particularly one as cognitively sophisticated as the dog. The current experiment adapted a paradigm from human infant studies to examine jealousy in domestic dogs. We found that dogs exhibited significantly more jealous behaviors (e.g., snapping, getting between the owner and object, pushing/touching the object/owner) when their owners displayed affectionate behaviors towards what appeared to be another dog as compared to nonsocial objects. These results lend support to the hypothesis that jealousy has some “primordial” form that exists in human infants and in at least one other social species besides humans.

## Introduction

In humans, jealousy is an emotion with far-reaching psychological and social consequences. For example, it typically emerges as the third leading cause of non-accidental homicide across cultures [1]. While the origins and possible function of jealousy have been debated, most theorists agree on one defining feature: It requires a social triangle, arising when an interloper threatens an important relationship. A common assumption has been that the elicitation of jealousy involves, and perhaps requires, complex cognitive abilities [2]–[3], including appraisals about the meaning of the rival threat to one's self (e.g., self-esteem) and to one's relationship. For example, Lewis [4] has proposed that the emergence of jealousy requires the cognitive ability to reflect on the self and to understand conscious intentions.

The vast majority of research in this area has concentrated on jealousy within romantic relationships particularly over potential or actual infidelity. Hence, functional or evolutionary analysis of jealousy has focused on the fitness consequences of loss of a romantic or sexual relationship (e.g., cuckoldry, loss of resources) and on the psychological and behavioral effects of protecting such relationships [5]–[7]. A broader functional view, however, would argue that jealousy evolved to secure resources not just in the context of sexual relationships, but also in any of a wide-range of valued relationships [1], [8]. Accordingly, the same underlying emotional process that gives rise to jealousy in sexual relationships also produces jealousy in other types of bonds (e.g., friendships).

One possibility is that jealousy first evolved in the context of sibling-parent relationships where dependent offspring compete for parental resources. An implication of this hypothesis is that jealousy may have a primordial or core form that can be triggered without complex cognition about the self or about the meaning of the social interaction [1], [9]. This primordial form of jealousy may be elicited by the relatively simple perception that an attachment figure or loved one's attention has been captured by a potential usurper, which suffices to elicit a motive to regain the loved one's attention and block the liaison. Primordial jealousy may serve as the building block for jealousy elicited by more complex cognitive processes. For example, in adult human relationships, the experience of jealousy is greatly impacted by additional appraisals about the meaning of the interaction (e.g., does this mean my mate will leave me? Am I unloveable?). In both primordial and complex cases of jealousy, there is a motivation to restore the relationship and remove the usurper. However, in the latter case, interpretations of the situation play a large role in the elicitation and experience of the emotion.

The theory that jealousy can take a primordial form finds support from the small but emerging body of research on human infant jealousy. Several studies [10]–[12] found that infants as young as 6-months of age show behaviors indicative of jealousy, for example, when their mothers interacted with what appeared to be another infant (but was actually a realistic looking doll). The infants did not display the same behaviors when their mothers attend to a nonsocial item (a book).

The functional account we are proposing would further predict that jealousy should occur not only in humans, but also in other social species in which emotional bonds between individuals develop and can be threatened by third parties. Interestingly, several observers of animal behavior, including Charles Darwin [13], have suggested that jealousy may exist in other species, particularly in dogs. This possibility has also been underscored in a recent paper that had owners recount specific cases of their animals displaying emotions [14]. Descriptions of dog jealousy were fairly consistent across owners and always involved a social triangle. When the owners gave attention and affection to another person or animal, the dogs seemed to engage in attention-seeking behaviors (pushing against the owner or in between the owner and the rival, barking/growling/whining) and some showed aggression. Reports of the occurrence of jealousy in dogs was at least as common, if not more so, than some other emotions that are often considered more primary (anxiety and anger). However, despite such reports, experimental evidence demonstrating behavioral indices of jealousy in dogs does not exist. This type of empirical research seems particularly important, given that dog owners also report that their animals experience guilt but experimental work raises some doubt about whether dogs do show guilt [15].

The idea that dogs are capable of jealousy is congenial to the burgeoning body of research on animal social cognition that reveals that dogs have sophisticated social-cognitive abilities. For example, dogs can use a variety of human communicative signals (e.g., pointing, eye gaze) to determine the location of hidden food [16], are better at using social cues than chimpanzees [16], show some sensitivity to reward inequity when a partner is rewarded and they are not [17] and appear aware of, and actively attempt to manipulate, the visual attention of their play partners [18].

### The Present Research

Although there are several reasons to predict that jealousy should lie within a dog's emotional repertoire, empirical evidence is lacking. The purpose of the present study was to construct a social situation and determine whether dogs, whose owners show affection to a potential interloper, engage in behaviors indicative of jealousy.

To evaluate dogs' jealous behaviors, we modified a paradigm used to assess jealousy in 6-month-old infants [10]–[12]. Thirty-six dogs were individually tested and videotaped while their owners ignored them and interacted with a series of three different objects. In the jealousy condition, the owner treated a stuffed dog, which briefly barked and wagged its tail, as if it were a real dog (e.g., petting, talking sweetly). In another condition, owners engaged in these same behaviors but did so towards a novel object (jack-o-lantern pail). This enabled us to test whether the elicitation of jealousy required that the owner show affection to an appropriate stimulus (what appeared to be a conspecific) or whether affectionate behaviors directed to a nonsocial stimulus would be enough to arouse jealous behaviors. In the third condition, the owner read aloud a children's book, which had pop-up pages and played melodies. This condition allowed us to test whether dogs' behaviors in the other conditions were indicative of jealousy per se (arising over the loss of affection and attention towards an interloper) or more general negative affect due to the loss of the owner's attention.

As discussed earlier, the proposed function of jealousy is to break-up a potentially threatening liaison and protect the primary relationship. This motivates several types of behaviors including approach actions such as attempts to get physically or psychologically between the attachment 3ure and the interloper, attending to the threatening interaction, seeking attention from the attachment figure, as well as indicators of negative emotion such as aggression, particularly toward the interloper [1]–[2], [8], [10]–[12], [19]. Across social species, we would expect to see similar types of behaviors that serve the function of this motivational state. Therefore, the specific behaviors assessed in our experiment were based on studies of jealousy in non-verbal human infants and adults as well as behaviors described by dog owners and experts as indicative of jealousy.

## Methods

### Subjects

This research was approved by the University of California San Diego Institutional Animal Care and Use Committee and by the University of California at San Diego Human Research Protections Program. Owners and pet dogs were recruited from the University of California, San Diego subject pool. Owners received partial course credit in psychology class for participation.

Dogs were tested individually at their homes and had to be less than 35 pounds or shorter than 15 inches. A size criterion was used because of the possibility that the jealousy manipulation would result in aggression and small dogs could be more easily controlled in such circumstances. Upon arrival at the subjects' homes, the experimenter obtained written consent from the dog owners for participation. The experimenter also gave a brief overview of the study stating, “For this experiment, we are examining individual differences in dog behavior across various situations. Thus, we'll be asking you to act out various situations in front of your dog, while ignoring him or her, while we film his or her reaction." The experimenter also said, "If you think that your dog will act aggressively in this situation, we ask that you and your dog don't participate, but you'll still receive your Experimetrix [course] credit." All owners chose to continue with the experiment.

The initial sample size was n = 37. However, one male dog was excluded from all analyses due to a miscommunication between the owner and experimenter resulting in one condition ending prematurely. Therefore, the final sample size was n = 36. The mean age of the dogs was 32.2 months (range = 4–135). Equal numbers of male and female dogs participated. The sample consisted of a variety of dog breeds as can be seen in Table 1. Owners (31 females and 5 males) had owned their dogs an average of 25.4 months (range  = 1–134).

**Table 1:** Dog Breeds.

Breed	*n*	(%)
Boston Terrier	1	(2.7)
Chihuahua	2	(5.4)
Dachshund	1	(2.7)
Havanese	1	(2.7)
Malinois	1	(2.7)
Maltese	3	(8.1)
Miniature Pincher	1	(2.7)
Miniature Schnauzer	1	(2.7)
Pomeranian	2	(5.4)
Pug	1	(2.7)
Shetland Sheepdog	1	(2.7)
Shih-tzu	2	(5.4)
Welsh Corgi	1	(2.7)
Yorkshire Terrier	3	(8.1)
Mix	14	(37.8)

### Procedure

Owners were not aware of the hypotheses of the experiment. A within-subjects design was employed with order counter-balanced across participants. Each experimental condition was videotaped and lasted for 1 minute, followed by a 30 sec post-condition phase in which the owner set the object down within reach of the dog. The owner completed a questionnaire after each condition and then the dog and owner were given one minute to freely interact in order to reduce any potential carryover effects from the previous condition.

### Conditions

#### Stuffed Dog

Owners were instructed to interact with a realistic-looking stuffed dog that barked, whined, and wagged its tail (which lasted for a total of approximately 8 secs) when a button on the top of its head was pressed. The owners were asked to press the button only once and to interact with the stuffed dog as if they were playing with a real dog. They were also instructed to completely ignore their dog, which was present in the room for the duration of the interaction.

#### Jack-o-lantern

In the novel object condition (jack-o-lantern), owners were given the same instructions as in the stuffed dog condition: to interact with a jack-o-lantern as if they were playing with a real dog.

#### Book

In the book control condition, owners were instructed to read aloud a children's book, which popped up and played melodies, as if they were reading to a young child. Total amount of time the object made a noise was closely matched to that of the dog condition (approximately 8 secs).

### Behaviors

Two raters, blind to the study's purpose, coded the videos. Unless otherwise noted, behaviors were coded as present or absent and are reported as percent of dogs showing such behaviors per condition. To compute interrater reliability, 29 dogs were coded by both raters. Behaviors in which interrater reliability was lower than .7 were not included in the analyses. For analyses that required whole numbers (i.e., Cochran's Test for presence/absence of a behavior), we used one rater's codes for half the dogs and the other rater's codes for the other half. Behaviors coded during the interaction of the owner with the object are described first, followed by the behaviors coded post-interaction.

#### Aggression

We coded for aggression given its link to jealousy in human adults [1] and in owners' reports of dog jealousy [14]. Our primary measure of aggression was whether the dog attempted to bite/snap at the object. Coders also attempted to code more subtle signs of aggression that include lip curling (lips being held open with force in order to expose teeth), raised tail–holding the tail up between the horizontal and vertical positions (an aggressive posture in wolves), and keeping the ears forward. Interrater reliability was low for ears forward (.57) and so this behavior was not analyzed. Our coders also reported difficulty assessing the raised tail in many dogs due to their physical characteristics (e.g., a clipped tail); however, interrater reliability was acceptable, so these analyses are reported in the results section. Lip curling was not seen across any condition.

#### Attention Seeking/Disruption of Interaction

The most common category of behavior in owner descriptions of their dogs' jealousy was attention seeking [14]. These behaviors often took the form of pushing the owner or attempting to get between the owner and the rival. We coded for both of these behaviors. Some owners also reported attempts by their dogs to shoo the rival away. In our coding scheme, we operationalized this as the dog pushing against the rival object. Another class of behaviors categorized as attention seeking by Morris et al. [14] was vocalizations (described as barking, growling, and whining), which we assessed. (Growling interrater reliability was lower than our .7 cutoff so was excluded from our analyses.)

#### Interest/Attention

The preverbal human infant literature (e.g., by Hart and colleagues) suggests that jealousy produces increased interest and attention toward the mother. We assessed several behaviors pertaining to attention, which included 1) looking at the owner, 2) looking at the rival object, 3) orienting away from the owner, and 4) orienting away from the object. The first two categories were operationally defined as having the head turned and gaze directed toward the owner/object; the later two were operationally defined as having the head and body turned away from the owner/object. Due to their frequency across conditions, a simple present/absent code for the entire interaction period did not adequately capture these behaviors. Therefore, we performed more fined grained coding that consisted of denoting whether the behavior was present or absent every 5 seconds, resulting in total score that ranged from 0–12. Total scores were then transformed into proportion of time behavior was present. (In the cases where both raters coded the same dog's behavior, the average of the two coders' scores was used for these measures.)

#### Behaviors coded during the 30-second post interaction period

During the post-interaction period, the owner put the object down and walked away. We coded four behaviors during this phase: 1) aggression/snapping directed at the object; 2) following the owner; 3) observing the object; and 4) ignoring the object. Due to the laborious nature of coding, we only coded for presence/absence of attention behaviors (observing/ignoring object) in this phase rather than assessing attention every 5 seconds as done in the condition phase. Additionally, in the dog condition, we noted if the dog sniffed the rear end of the toy dog. This was included as a measure of whether the dog perceived the stuffed dog as a real dog.

#### Additional exploratory measures

In addition to assessing jealousy, we used this opportunity to explore attachment style in dogs (and its possible interaction with our conditions), which we mention here for the sake of completeness. We created a scale for owners to complete regarding their dog's attachment style and coded for behaviors that might be linked to different attachment styles. These included behaviors that might be indicative of anxiety (paw slightly raised; yawning) and of submission (ears back, tail down, and licking). We also attempted to code tail wagging to the left vs. the right because work by Quaranta, Siniscalchi, and Vallortigara [20] suggested that the former is associated with withdrawal and the latter with approach. However, our coders found this too difficult to assess via video. Analyses pertaining to attachment style are not reported here given their exploratory nature and the difficulty of measuring some of these behaviors.

## Results & Discussion

**Figure 1:**
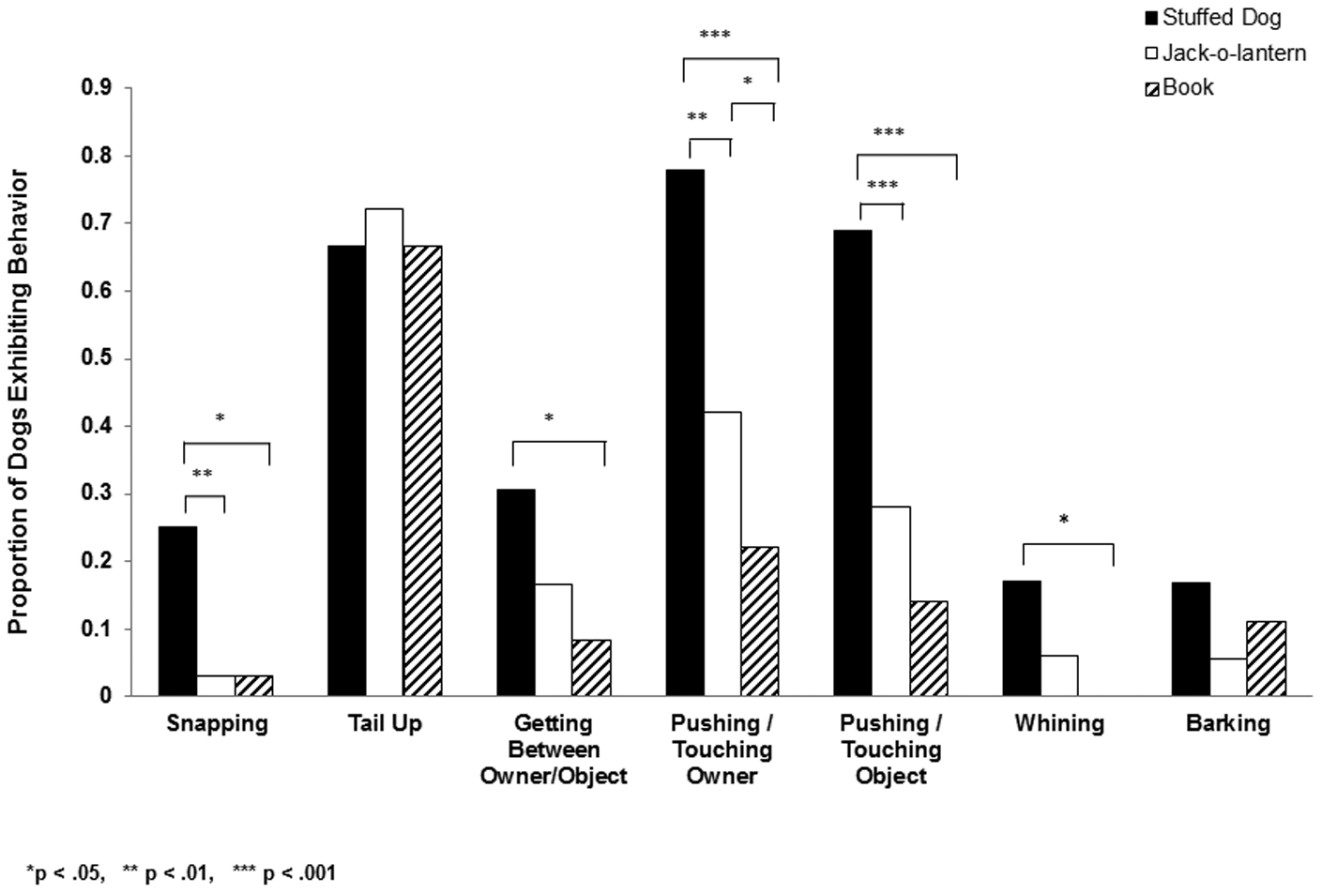
Comparisons of the proportion of dogs exhibiting each type of behavior in each of the three experimental conditions.

Figure 1 shows the proportion of dogs that engaged in the various behaviors during the interactions with the objects in each condition. Our analyses showed significantly greater aggression in the jealousy condition, Cochran's Q, χ^2^ (2) = 12.80, p< .002. One fourth of the dogs snapped at the object in the jealousy condition but only 1 dog did so in the other two conditions. (Results from follow up McNemar nonparametric tests are presented in Figure 1.) We, however, did not see a difference across conditions in the number of dogs that placed their tails between the vertical and horizontal positions (Cochran's Q, χ^2^ (2) =  .67, *ns*). This may be due to our coders having difficulty accurately assessing tail movement (e.g., in dogs with snipped tails). In the post-interaction phase (when the owner had put the object down), 36% of the dogs snapped at the stuffed dog while snapping behavior in the other conditions was confined to one dog (Cochran's Q, χ^2^ (2) = 20.57, p< .001). The aggression in the jealousy condition is particularly impressive given that we only tested dogs whose owners believed that their dogs would not behave aggressively in novel situations.

The next series of analyses examined attention seeking and specifically focused on the behaviors most commonly noted by owners when describing their animals' jealous behaviors [14]. Dogs were significantly more likely to push or touch their owners (Cochran's Q, χ^2^ (2) = 26.87, p< .001) and the object (Cochran's Q, χ^2^ (2) = 24.07, p< .001) in the jealousy condition relative to either the jack-o-lantern or book conditions. Results for follow up McNemar nonparametric tests are presented in Figure 1. Of particular interest, dogs specifically tried to get between the owner and the object more often in the jealousy condition (Cochran's Q, χ^2^ (2) = 6.53, p< .04). Such behaviors aimed at preventing or breaking up a liaison have been hypothesized to be the primary motivational state that accompanies jealousy and that distinguishes jealousy from other emotions such as anger [1], [19]. Although vocalizations during the experiment were relatively infrequent, whining occurred significantly more in the jealousy condition than in the book condition. Barking did not significantly differ across conditions.

At the suggestion of a reviewer, we summed the behaviors presented in Figure 1 for each condition in order to get a sense of the effect size of the jealousy condition and performed within-subjects ANOVA with partial eta-squared tests. Partial eta-squared for the dog vs. jack-o-lantern conditions was .41 and for dog vs. book was .58.

**Figure 2:**
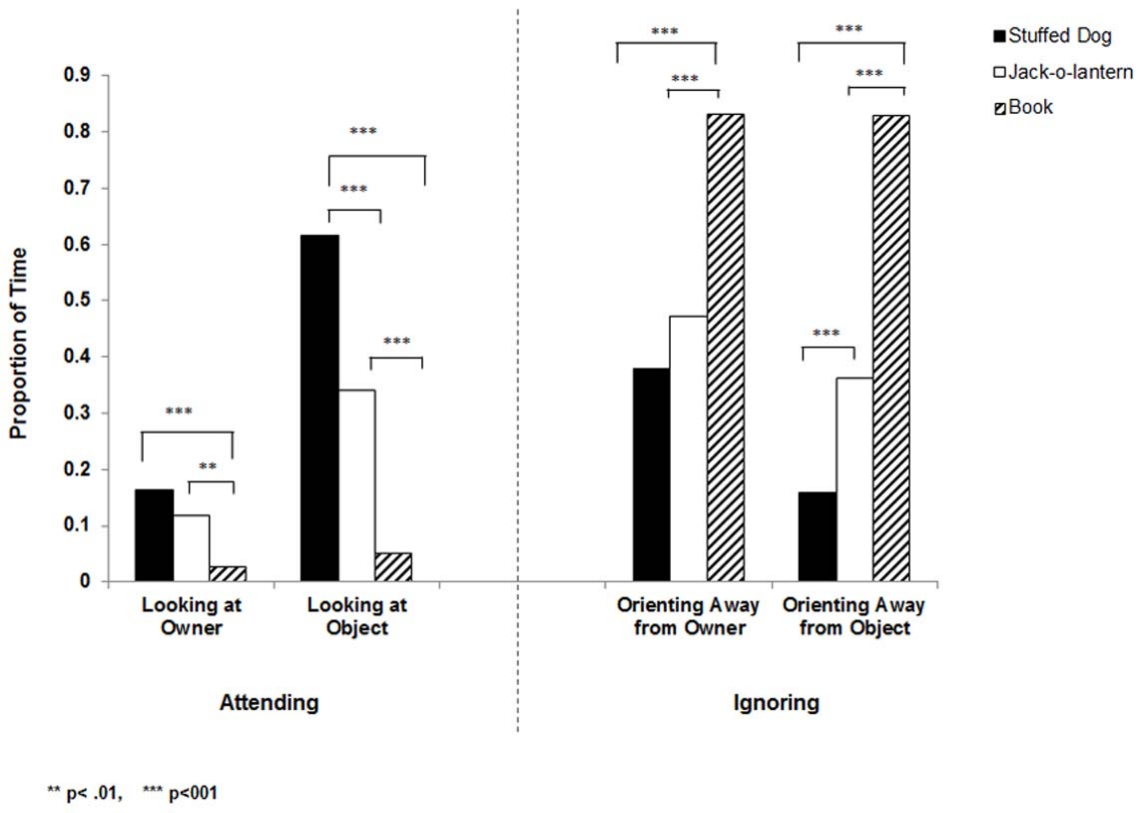
Proportion of time dogs spent attending to and orienting away from their owner and the object in each of the three experimental conditions.

In the preverbal human infant literature, jealousy is accompanied not only by negative affect but also by heightened interest and attention toward the mother while she is interacting with what appears to be another infant [10]–[12]. We found similar effects in dogs. Results for within-subject ANOVAs are presented in the text while results for follow up paired t-tests are displayed in Figure 2. As shown in Figure 2, dogs gazed significantly more at their owners (*F*(2, 70) = 7.47, p< .001, 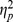  =  .18) and the objects (*F*(2, 70) = 56.39, *p*< .001, 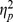  =  .62) in the conditions in which the owner was displaying affection towards an object relative to the control condition of reading aloud. Furthermore, when the object of affection appeared to be a conspecific, dogs looked more at the object relative to when it was a novel item (jack-o-lantern). This pattern is furthered supported by the inversely related measures of disinterest, operationally defined by orienting the body away from the owner and object (Figure 2). Dogs spent significantly more time ignoring the owner and object in the book condition relative to the conditions that involved displays of affection: *F*(2, 70) = 30.62, *p*< .001, 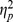  =  .47 for owner; *F*(2, 70) = 87.27, *p*< .001, 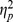  =  .71 for object.

Behaviors during the post-task period were similar to those seen during the actual interaction (although less informative given that behavior was merely coded as present/absent). More dogs ignored the object in the jack-o-lantern (94.4%) and book (91.7%) conditions than in the dog condition (52.8%); Cochran's Q, χ^2^ (2) = 20.01, p< .001. There was also a significant difference in the number of dogs who observed the object across conditions (72.2% in dog condition; 63.9% in jack-o-lantern condition; and 44.4% in book condition): Cochran's Q, χ^2^ (2) = 6.58, p< .04. There was not a significant difference in the number of dogs who followed their owner after each condition, χ^2^ (2) = 4.67, p =  .10.

### Was the stuffed dog perceived as real?

One might wonder whether the subject dogs believed that the stuffed animal was a real dog. The data discussed previously, particularly the aggressive behaviors aimed at the stuffed animal, would seem to suggest that they did. Perhaps even more compellingly, 86% of the dogs sniffed the anal region of the toy dog during the experiment or post-experiment phases. Having a faux rival dog enabled us to maximize the amount of control we had in the experiment. That jealousy behaviors were seen under these somewhat impoverished social conditions leads us to predict that such behaviors would be even more enhanced in the present of an actual rival dog who responded to the owner's affectionate displays.

In the present work any single behavior might not be indicative of jealousy per se. However, the pattern of behaviors, particularly when dogs were confronted with their owners displaying affection to what appeared to be another dog, is similar to the constellation of behaviors seen in humans. These data thus present a strong case that domestic dogs have a form of jealousy.

### Individual Differences

An anonymous reviewer suggested that our data might reflect individual differences in dog cognition and/or jealousy. One possibility is that only some dogs perceived the stuffed dog as real and that this difference in cognitive ability resulted in jealousy in some dogs but not others. To the reviewer's mind, only those dogs that aggressed against the “rival” dog can be shown to definitively consider the fake dog stimulus as real. We believe that the fact that the vast majority of the dogs sniffed the anal region of the stuffed dog (including all of the aggressive dogs) suggests that most of the dogs conceptualized the dog as real, but we cannot rule out the reviewer's suggestion.

However, even if most dogs perceived the stimulus as real, it is still possible that only some of them reacted with jealousy to it. The reviewer suggested some additional analyses to explore possible individual differences. One question is whether the snapping dogs were the only ones to show additional jealous behaviors. As described below, our data suggest that they were not, although their jealousy might be argued to be more intense in some respects than their nonsnapping peers. In total, 41.7% of dogs snapped during or after their owner interacted with the stuffed dog. We found that these aggressive dogs did display many other jealous behaviors: All of them pushed at the owner and 86.7% pushed at the fake dog during the jealousy interaction. There was also some tendency for these dogs to spend more time (although not significantly so) attending to the owner and the fake dog (and conversely less time ignoring the owner and rival) than nonsnapping dogs. However, not all jealous behaviors were more common in the snapping dogs. Whining was similar across the two types of dogs and while 26.7% of the snapping dogs attempted to get between their owner and the rival dog, 33.3% of the nonaggressive dogs did so. Moreover, many of the nonsnapping dogs also displayed other jealousy indicators. For example, 61.9% pushed at the owner and 57.1% pushed at the stuffed dog. These percents are higher if the dogs that did not sniff the rear end are excluded (who likely did not perceive the stuffed dog as a threat). Thus, these exploratory analyses would argue against the suggestion that only aggressive dogs displayed jealous behaviors, although their jealousy may be viewed as more extreme. These data raise the possibility that dogs, like humans, show individual differences in how jealousy is exhibited, which ranges from attention seeking and restorative behaviors to aggressive acts.

The fact some dogs (13.8%) failed to sniff the rear end of the fake dog raises stimulating questions for future studies. Jealous behaviors were infrequent among these animals, suggesting that they were not in an emotional state (e.g., none pushed on the owner or the stuffed dog and only one got between them). One interesting possible avenue for future work is to examine the cognitive abilities that are associated with not believing the stuffed dog is real. It might be that such dogs are less cognitively sophisticated (they do not perceive the toy as representation of a real dog.). However, it is also possible that they are more sophisticated, (i.e., were not fooled into believing that the stuffed dog was genuine, and hence it was not a threat).

In sum, it may be that while all dogs have the neurobiological cognitive capacity for jealousy, the current situation may have failed to induce the emotional state in some dogs. Understanding what factors (cognition or social dynamics) contribute to individual differences in dog jealousy would seem a ripe area for future work.

### Concluding Remarks

The current findings add support to the emerging view [1], [9] that there is a primordial form of jealousy. This emotional state does not presuppose complex interpretations of the behavior of the rival and the attachment figure and its meaning to the self, (although such cognitions clearly can impact jealousy in adult interpersonal relationships). Primordial jealousy appears not only ontogenetically early in humans but may also have emerged phylogenetically early. We use the term ‘primordial’ to reflect a state that motivates jealous action tendencies that are similar across dogs and humans (of course, these findings do not speak to whether the subjective experience of the emotional state is similar).

If jealousy is phylogenetically old, then one might expect to see some form of it in other animal species that form emotional bonds that can be threatened by rivals, whether that rival be a sibling, or a mate, or another group member. Here we tentatively suggest some possibilities about features that might have given rise to jealousy, which could be explored in further work.

One possibility is that jealousy evolved in species that have multiple dependent young that concurrently compete for parental resources such as food, attention, care, and affection. It is easy to imagine the advantages that might be gained by a young animal that is not only alert to interactions between siblings and parents, but also motivated to interpose itself in such interactions. Several of the behaviors assessed in the current work (e.g., pushing on the owner, getting between the owner and “rival” dog) would seem to serve that goal. Future work might look at how effective these behaviors are in natural triadic interactions (e.g., does pushing on one's mother divert her attention from a sibling?). Another possibility is that jealousy behaviors emerged to protect pair-bonded sexual relationships from interlopers. If so, then jealousy might not exist in species that do not pair-bond, regardless of the number of offspring reared simultaneously. (To date, little is known about the relationship between romantic and nonromantic jealousy.) Finally, it is possible that jealousy evolved in animals that require cooperation from other group members for survival and in which alliances are formed (and therefore, can be threatened by rivals). These possibilities are difficult to tease apart by studying dogs given that they are litter born, have the potential for pair-bonding (see Bradshaw [21] for discussion), and hunt cooperatively. It would be informative to examine jealousy in species that differ on these factors (e.g., domestic cats, which bear litters but are not pair-bonded).

Finally, it is also possible that the long co-evolution and domestication of dogs, which likely gave rise to many of their remarkable social-communicative skills [16], [22], created their capacity for jealousy. (Perhaps this is a function of their emotional bonding with humans along with their motivation and ability to track human gaze/attention. Humans, afterall, have been rich resource providers over our coevolution.) One might speculate that even if several social species have the capacity for jealousy, dogs may be the only species besides humans in which the emotion can be evoked in connection with a member of a different species. Future studies that examine the affective and cognitive abilities of a variety of animal species could help tease apart these various intriguing possibilities. Such work is particularly warranted given that a large percentage of owners of some other types of domestic animals such as horses, birds, and cats also report signs of jealousy in their animals [14]. Moreover, some of these species such as horses have been shown to be highly sensitive to human attentional cues [23]. Further research on the neurobiological components of and influences on emotions in both humans and other animals may also help disentangle the similarities and differences of emotion and social behavior across species [24]–[25].

In closing, these findings add additional support to the view that jealousy can arise in the absence of complex interpretations of the meaning of the rival and loved one's interaction and occurs in another species besides humans. We hope the current work will inspire further research into the social emotions of animals.
